# Negative response of photosynthesis to natural and projected high seawater temperatures estimated by pulse amplitude modulation fluorometry in a temperate coral

**DOI:** 10.3389/fphys.2015.00317

**Published:** 2015-11-04

**Authors:** Erik Caroselli, Giuseppe Falini, Stefano Goffredo, Zvy Dubinsky, Oren Levy

**Affiliations:** ^1^Marine Science Group, Department of Biological, Geological and Environmental Sciences, University of BolognaBologna, Italy; ^2^Department of Chemistry <<G. Ciamician>>, University of BolognaBologna, Italy; ^3^The Mina and Everard Goodman Faculty of Life Sciences, Bar-Ilan UniversityRamat-Gan, Israel

**Keywords:** pulse amplitude modulation fluorometry, global warming, scleractinian, *Balanophyllia europaea*, photosynthetic efficiency, zooxanthellae

## Abstract

*Balanophyllia europaea* is a shallow water solitary zooxanthellate coral, endemic to the Mediterranean Sea. Extensive field studies across a latitudinal temperature gradient highlight detrimental effects of rising temperatures on its growth, demography, and skeletal characteristics, suggesting that depression of photosynthesis at high temperatures might cause these negative effects. Here we test this hypothesis by analyzing, by means of pulse amplitude modulation fluorometry, the photosynthetic efficiency of *B. europaea* specimens exposed in aquaria to the annual range of temperatures experienced in the field (13, 18, and 28°C), and two extreme temperatures expected for 2100 as a consequence of global warming (29 and 32°C). The indicators of photosynthetic performance analyzed (maximum and effective quantum yield) showed that maximum efficiency was reached at 20.0–21.6°C, slightly higher than the annual mean temperature in the field (18°C). Photosynthetic efficiency decreased from 20.0 to 13°C and even more strongly from 21.6 to 32°C. An unusual form of bleaching was observed, with a maximum zooxanthellae density at 18°C that strongly decreased from 18 to 32°C. Chlorophyll *a* concentration per zooxanthellae cell showed an opposite trend as it was minimal at 18°C and increased from 18 to 32°C. Since the areal chlorophyll concentration is the product of the zooxanthellae density and its cellular content, these trends resulted in a homogeneous chlorophyll concentration per coral surface across temperature treatments. This confirms that *B. europaea* photosynthesis is progressively depressed at temperatures >21.6°C, supporting previous hypotheses raised by the studies on growth and demography of this species. This study also confirms the threats posed to this species by the ongoing seawater warming.

## Introduction

Reef-building corals are highly dependent on their symbiotic relationship with photosynthetic dinoflagellates of the genus *Symbiodinium*, commonly referred to as zooxanthellae (Brandt, 1881). As part of this mutualistic endosymbiosis, several zooxanthellate corals receive most of their carbon and energy requirements from their symbionts' photosynthesis (Falkowski et al., 1984, 1993; Muscatine et al., 1984; Tremblay et al., 2012). During the last three decades, several studies worldwide report cases of disruption of this symbiosis causing mass bleaching events, which render corals white through the loss of symbionts or pigments within them (coral paling). The loss of zooxanthellae, due to environmental stresses, impacts coral energy and carbon budget, and may result in death if the stress is severe and prolonged (Glynn, 1996; Lesser, 2011), unless the symbiosis is re-established from remaining zooxanthellae (Koren et al., 2008). The main trigger for these bleaching events is elevated temperature acting synergistically with high irradiance (Brown, 1997; Dunne and Brown, 2001; Fitt et al., 2001; Jones and Hoegh-Guldberg, 2001; Lesser and Farrell, 2004) and UV radiation (Iluz et al., 2008).

High temperature has been shown to adversely affect the host (Lasker et al., 1984; Glynn et al., 1985; Porter et al., 1989; Gates et al., 1992; Brown and Cossins, 2011), but the algal symbionts seem less tolerant to heat stress than their coral hosts, so that it is generally accepted that damage to algal photosynthetic apparatus (causing oxidative stress; Lesser, 2003) is the primary step of the bleaching process (Jones et al., 1998; Warner et al., 1999; Tchernov et al., 2004; Smith et al., 2005). Zooxanthellae can regulate excess excitation energy via photoprotective non-photochemical quenching, associated with xanthophyll cycle-dependent thermal energy dissipation of excess light via the de-epoxidation of the xanthophyll carotenoid diadinoxanthin to diatoxantin (Ambarsari et al., 1997; Brown et al., 1999). Moreover, both symbiotic partners have protective mechanisms against oxidative cellular damage, such as antioxidant enzymes, heat shock proteins (e.g., Downs et al., 2002; Richier et al., 2005; Levy et al., 2006) and mycosporine-like amino acids (Dunlap and Shick, 1998; Yakovleva et al., 2004). Despite the mass of studies on tropical corals documenting both positive (Jacques et al., 1983) and negative (Jones et al., 2000; Nakamura et al., 2003) effects of elevated temperatures on photosynthesis, few studies have investigated the response of temperate coral symbionts (Jacques et al., 1983; Ben-Haim et al., 1999; Jones et al., 2000; Nakamura et al., 2003; Rodolfo-Metalpa et al., 2006).

*Balanophyllia europaea* is a solitary and zooxanthellate scleractinian, endemic to the Mediterranean Sea where it colonizes rocky substrates (Zibrowius, 1980, 1983). Because of its symbiosis with zooxanthellae, it is constrained to illuminated shallow waters, down to 50 m depth (Zibrowius, 1980). Along the Italian coastline, its net calcification rate is negatively correlated with sea surface temperature (SST; Goffredo et al., 2009), resulting in a progressive decrease of skeletal bulk density (Goffredo et al., 2007) and an increase in skeletal porosity (Caroselli et al., 2011), especially of larger sized pores (Fantazzini et al., 2013). This determines a decrease of the resistance of the skeleton to mechanical stress (Goffredo et al., 2015). Furthermore, its population stability and abundance (which can reach hundreds of individuals per m^2^) decrease with increasing SST, as evidenced by a progressive lack of juveniles (Goffredo et al., 2007, 2008). It has been proposed that the negative effects of temperature (Goffredo et al., 2007, 2008, 2009) may be caused by a reduction of photosynthesis, causing a consequent decrease of energetic resources for all metabolic processes of the host.

Given the threats for the survival of this species in light of global temperature increase (Goffredo et al., 2008, 2009; Caroselli et al., 2011), the present study aims at analyzing for the first time the response of photosynthesis of the symbionts of *B. europaea* to the whole range of temperatures naturally experienced in the field and projected for the next future, to verify if photosynthesis is depressed at high temperatures.

## Materials and methods

### Sample collection

**Figure 1:**
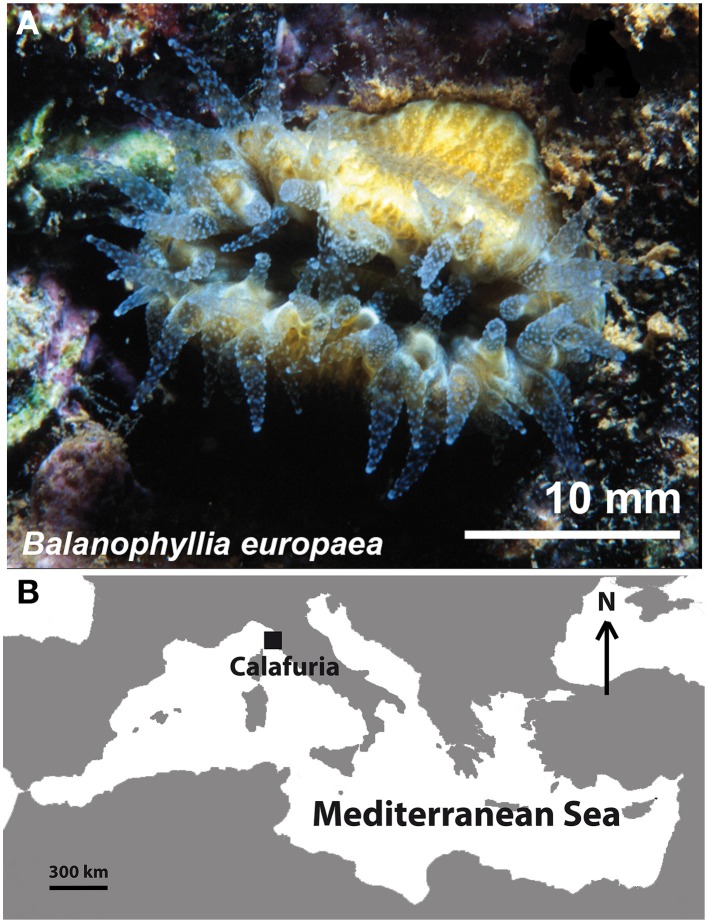
***Balanophyllia europaea***. Living specimen **(A)** and map **(B)** of the Mediterranean Sea indicating the Calafuria site where corals were collected (43°27′N, 10°21′E).

One hundred specimens of *B. europaea* (Risso, 1826) were randomly collected at 5–7 m depth at Calafuria, Italy, on 30th July 2009 (Figure 1). Corals were immediately taken to the aquarium system of the Department of Biological, Geological and Environmental Sciences of the University of Bologna (Italy), and housed in a tank with artifical seawater at constant temperature (18°C, equal to Calafuria seawater temperature at time of collection). Corals were allowed to recover for 15 days. During this period, corals were fed three times per week with *Artemia salina* nauplii, and aquarium lights were set to match the seasonal photoperiod (16 h of light, 8 h of dark) and light intensity (PAR = 450 μmol photons m^−2^ s^−1^) at 6 m depth in Calafuria, at 1200 h with clear skies. Corals were then shipped to the aquarium system of the Bar-Ilan University, Ramat-Gan (Israel) after obtaining the required CITES permit (CITES n° IT/EX/2009/MCE/00086; 2009/43407), where they were randomly separated into five subsets and housed in five tanks with the same environmental settings used in the Bologna aquarium. Polyps were allowed to recover from shipment for 15 days, until they appeared healthy and fully expanded their tentacles at night.

### Experimental setup

Five tanks were used for the experiment, named by the five temperature treatments (Tank 13, initally housing 19 corals; Tank 18, initially housing 12 corals; Tank 28, initially housing 15 corals; Tank 29, initially housing 19 corals; Tank 32, initially housing 35 corals). Tank 32 housed more corals than the other tanks because we expected higher mortality at this high temperature than in other treatments. Temperature was controlled using heaters or a refrigerating system connected to electronic controllers. Starting from experimental day 0, temperature of each tank was adjusted at steps of 1°C per day, until the tank reached the treatment temperature (Table 1). Temperature treatments were selected as (1) minimum annual temperature at Calafuria (13°C, in Tank 13); (2) mean annual temperature at Calafuria (18°C, in Tank 18); (3) maximum annual temperature at Calafuria (28°C, in Tank 28); (4) maximum annual temperature under an optimistic scenario (RCP2.6) of +1°C (29°C, in Tank 29) by the Intergovernemental Panel on Climate Change (IPCC); and (5) maximum annual temperature under a pessimistic IPCC scenario (RCP8.5) of +4°C (32°C, in Tank 32). Corals were regularly fed three times per week with *A. salina* nauplii throughout the experiment. Salinity was measured each day and kept at 38 ppt. Water pH was measured every 3 days using a pH electrode and was stable at 8.3 (no correction was neeeded during the experiment). Nitrite and ammonium concentrations were measured every 3 days with commercial aquarium kits (Tetra, Melle, Germany) and never reached values requiring corrections. After each Tank reached its treatment temperature (day 0 for Tank 18, day 5 for Tank 13, day 10 for Tank 28, day 11 for Tank 29, and day 14 for Tank 32; gray areas in Table 1), 12–15 corals were randomly taken from each tank during different days and their photosynthetic rates and related parameters were determined (see below). Some corals were also randomly taken and analyzed at intermediate temperature steps with respect to the five temperature treatments, to have some additional information along the entire range of temperatures from 13 to 32°C (Table 1).

**Table 1:** **Temperature (T) inside each tank at the end of each day of experiment**.

**Day**	**Tank 13 T (°C)**	**Tank 18 (°C)**	**Tank 28 (°C)**	**Tank 29 (°C)**	**Tank 32 (°C)**
0	18	18^*^	18	18	18
1	17	18	19	19	19^*^
2	16	18	20	20^*^	20
3	15^*†^	18^*^	21	21	21
4	14^*^	18	22	22	22^*^
5	13^*^	18	23	23	23
6	13	18	24	24	24
7	13	18	25	25	25
8	13	18	26	26	26
9	13	18	27	27	27^*^
10	13	18	28^*^	28	28
11	13	18	28^*^	29^*^	29
12	13	18	28^*^	29^*^	30
13	13	18	28	29	31
14	13	18	28^*^	29^*^	32^†^
15	13	18^*^	28	29	32^*^
16	13^*^	18	28	29	32^*^
17	13	18	28	29^*^	32^*^
18	13	18	28	29	32^*††^
19	13	18	28	29	32^†^
20	13	18	28	29	32^†^
21	13	18	28	29	32^††^
22	13	18	28	29	32^††^
23	13^*^	18	28	29	32
24	13	18	28	29	32
25	13	18^*^	28	29	32^*†^
26	13	18	28	29	32
27	13	18	28	29	32
28	13	18	28	29	32
29	13	18	28	29	32
30	13^*^	18	28^*^	29	32
31	13	18	28	29^*^	32

*Each tank was named after the temperature treatment (indicated as gray areas). One asterisk means that three corals were collected from that tank for analyses. One symbol ^†^ appears for each coral that died in that tank that day*.

### PAM fluorometry

Fluorometry is a method for estimating photosynthetic activity from the fluorescent properties of chlorophyll *in vivo* (Maxwell and Johnson, 2000). Variable fluorometry measurement of zooxanthellae is commonly used as a coral health proxy (Warner et al., 1999; Bhagooli and Hidaka, 2003; Suwa et al., 2008). Each sample was analyzed with all the procedures to estimate the parameters described in the following sections. All PAM fluorometry analyses were performed at 1030 h.

#### *F*_*v*_/*F*_*m*_

*F*_*v*_/*F*_*m*_ is a proxy for photochemical efficiency and a relative measure of the rate at which PS II can use light to process electrons flowing during photosynthesis and the photosynthetic efficiency of light reactions (Hoegh-Guldberg and Jones, 1999). *F*_*v*_/*F*_*m*_ was measured using an Imaging-PAM (MAXI Imaging-PAM, Walz, Effeltrich, Germany) on corals submerged in a shallow bath of seawater taken by their housing tank. Using the software ImagingWin v 2.32 (Walz, Effeltrich, Germany), for each polyp, one “area of interest” (AOI) for measuring *F*_*v*_/*F*_*m*_ was determined on the oral region of the polyp. To account for any potential effects of micro-scale heterogeneity of the fluorescence signal due to differences between types of coral tissues, each AOI included the mouth, tentacles, and corallite wall tissue (see Kühl et al., 1995; Ralph et al., 2002, 2005; Hill et al., 2004; Ulstrup et al., 2006). Care was taken to leave only a very thin layer of water above the AOI to minimize measurement errors. The Imaging-PAM was used to analyze the photosynthetic efficiency of corals with the following settings: Measuring Intensity = 3, Measuring Frequency = 1, Actinic Light Intensity = 7, Actinic Width = 0, Image Correction = off, Gain = 4, Damping = 2, Saturating Intensity = 10, Red Gain = 30, Red Intensity = 3, NIR = 9, *F*_*m*_-Factor = 1, *F*-Factor = 1. Corals were dark-adapted for 15 min prior to measuring *F*_*v*_/*F*_*m*_ (Warner et al., 1996). The Imaging-PAM saturating pulse in the dark yielded minimum fluorescence, *F*_0_, and maximum fluorescence, *F*_*m*_. From these parameters, *F*_*v*_/*F*_*m*_ was calculated (Schreiber, 2004):

(1) $$\begin{array}{r} F_{v}∕F_{m}=\left(F_{m}-F_{0}\right)∕F_{m} \end{array}$$

#### Rapid light curves

Rapid light curves (RLCs; White and Critchley, 1999; Ralph and Gademann, 2005) were obtained with the Imaging-PAM right after *F*_*v*_/*F*_*m*_ determination, and comprised quantum yields at 15 incremental irradiance steps (0, 1, 11, 21, 36, 56, 81, 111, 146, 186, 231, 281, 396, 531, 701, 1076 μmol photons m^−2^ s^−1^) of 1 min duration each. At the end of each irradiance step a saturating pulse was given by the Imaging-PAM producing a minimum fluorescence, *F*, and maximum fluorescence, $F_{m}\prime$, in the light. From these parameters effective quantum yield (Δ*F*/$F_{m}\prime$) was calculated:

(2) $$\Delta F/F_{m}\prime =(F_{m}\prime -F)/F_{m}\prime$$

Δ*F*/$F_{m}\prime$ values at the irradiance step of 396 μmol photons m^−2^ s^−1^ (Δ*F*/$F_{m}\prime$_396_) were derived from the RLCs and compared among temperature treatments, since this PAR step was the closest to the maximum light levels recorded at site and depth of collection (450 μmol photons m^−2^ s^−1^). RLCs not only show the light-acclimation state over the past few minutes, but are also strongly influenced by long-term light exposure, providing quantitative insight into the light acclimatization of corals at different temperatures (Ralph and Gademann, 2005).

#### Non-photochemical quenching

Non-photochemical quenching (NPQ) was measured with the Imaging-PAM along the RLC for all samples using the following equation (Maxwell et al., 1995; Schreiber, 2004):

(3) $$\text{NPQ}=(F_{m}-F_{m}\prime )/F_{m}\prime$$

The parameter normally shows a dose-dependent response (Ralph and Gademann, 2005) until steady-state takes place. NPQ describes the magnitude of non-photochemical processes, mainly xanthophyll-cycle mediated thermal emission (Maxwell et al., 1995; Pinchasov-Grinblat et al., 2013). NPQ values at the irradiance step of 396 μmol photons m^−2^ s^−1^ (NPQ_396_) were derived from the RLCs and compared among temperature treatments.

### Zooxanthellae and chlorophyll quantification

Immediately after PAM analyses, coral tissue of each sample was removed from the skeleton using an airbrush with filtered artificial seawater (FSW). The slurry was homogenized on ice with an electric homogenizer and centrifuged at 5000 rpm for 5 min at 4°C. The resulting zooxanthellae pellet was separated from the supernatant (host tissue) and resuspended in 2 ml FSW, centrifuged and resuspended two more times, thus obtaining all the zooxanthellae of the coral suspended in 2 ml. A 0.5 ml subsample was used for zooxanthellae count, and a 1 ml subsample for chlorophyll *a* (chl *a*) measurement.

Zooxanthellae counts were performed using a Neubauer haemocytometer. The mitotic index was determined from the number of cells appearing as doublets during the counts (Jones and Yellowlees, 1997). For quantification of chl *a*, the 1 ml subsample was centrifuged at 5000 rpm for 5 min at 4°C and the supernatant discarded. One ml of acetone 90% was added to the pellet and the sample vial was kept in the dark overnight at 4°C to extract the pigments. Chl *a* concentration was determined spectrophotometrically (Jeffrey and Humphrey, 1975). Zooxanthellae density (cells mm^−2^) and chl *a* concentration (pg mm^−2^) were normalized to coral surface area, which was determined using the paraffin wax technique (Stimson and Kinzie, 1991). Cellular chlorophyll content (pg zooxanthellae cell^−1^) was also calculated.

### Statistical analyses

One-way analysis of variance (ANOVA) was used to compare the mean analysis day, *F*_*v*_/*F*_*m*_, NPQ_396_,zooxanthellae number per area, and mitotic index among temperature treatments, after checking for variance homogeneity with a Levene's test.

When ANOVA assumptions were not met, the rank-based, non-parametric Kruskal–Wallis test was used to compare mean Δ*F*/$F_{m}\prime$_(396)_, *chl a* content per zooxanthellae cell, and *chl a* content per area among temperature treatments. This distribution-free test is more robust than ANOVA in the case of a non-normal distribution of sample data, and it is a viable alternative to parametric statistics (Potvin and Roff, 1993).

The relationships between *F*_*v*_/*F*_*m*_, Δ*F*/$F_{m}\prime$_(396)_, NPQ_396_, *chl a* content per zooxanthellae cell, and *chl a* content per area, mitotic index and temperature was fitted with a quadratic function and analyzed with a polynomial regression analysis. All analyses were computed using PASW 18 (IBM, Armonk, NY, USA).

## Results

Mean analysis day (Table 1) was homogeneous among the five temperature treatments (ANOVA, *P* > 0.05).

**Figure 2:**
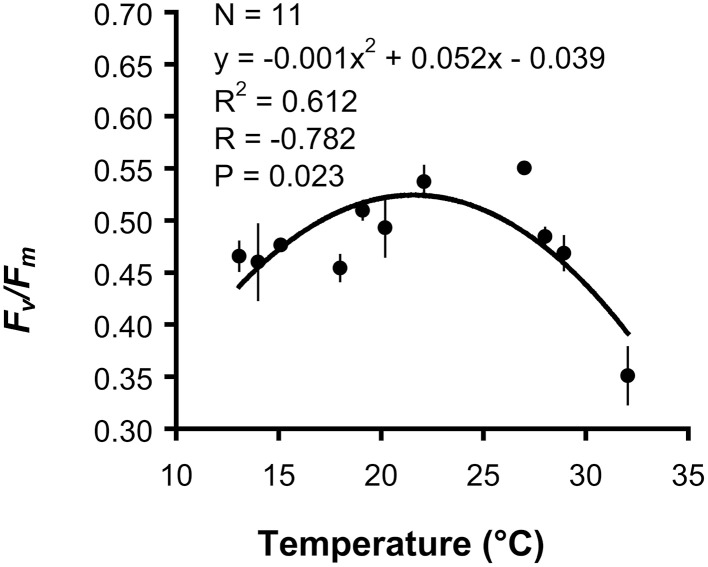
*****Balanophyllia europaea*****. Regression and correlation analysis between mean maximum quantum yield (*F*_*v*_*/F*_*m*_) and temperature using a quadratic function model. Error bars represent the standard error. N number of temperature treatments.

Mean *F*_*v*_/*F*_*m*_ was significantly different among temperature treatments (ANOVA, *P* < 0.001). The maximum value was obtained at 27°C, with a mean *F*_*v*_/*F*_*m*_ of 0.550 (Table 2). When fitted with a quadratic function, mean *F*_*v*_/*F*_*m*_ was correlated with temperature, whose variation explained 61% of *F*_*v*_/*F*_*m*_ variance. According to the quadratic function, maximum *F*_*v*_/*F*_*m*_ occurred at 21.6°C and while *F*_*v*_/*F*_*m*_ decreased by 17.2% from 21.6 to 13°C, it decreased by 23.7% from 21.6 to 32°C (Figure 2).

**Table 2:** **Number of analyzed corals (n), mean ***F***_***v***_/***F***_***m***_, Δ***F***/***F_m_′***_(396)_, NPQ_396_, number of zooxanthellae per area, ***chl a*** content per zooxanthellae cell, ***chl a*** content per area, and mitotic index for each temperature**.

**Temperature (°C)**	***n***	***F_*v*_*/*F_*m*_***	**Δ*F*/*F_m_*′_(396)_**	**NPQ_396_**	**Zoox number (n° mm^−2^)**	***chl a* per zoox cell (pg cell^−1^)**	***chl a* per area (pg mm^−2^)**	**Mitotic index**
13	12	0.466 (0.049)	0.199 (0.020)	0.55 (0.11)	8178 (3314)	1.21 (0.99)	8012 (3587)	0.071 (0.016)
14	3	0.460 (0.063)	0.194 (0.031)	0.69 (0.08)	11,227 (5126)	0.90 (0.38)	8792 (956)	0.059 (0.012)
15	3	0.476 (0.005)	0.204 (0.011)	0.55 (0.08)	10,567 (3797)	0.75 (0.28)	7250 (387)	0.069 (0.009)
18	12	0.454 (0.044)	0.190 (0.017)	0.61 (0.11)	7991 (2708)	0.93 (0.48)	6945 (2847)	0.102 (0.038)
19	3	0.510 (0.015)	0.208 (0.008)	0.58 (0.10)	13,983 (2698)	0.50 (0.16)	6924 (2686)	0.152 (0.021)
20	3	0.493 (0.048)	0.213 (0.036)	0.54 (0.11)	11,549 (2387)	0.57 (0.03)	6540 (1139)	0.148 (0.015)
22	3	0.537 (0.026)	0.211 (0.009)	0.61 (0.04)	11,388 (1435)	0.80 (0.20)	9296 (3391)	0.171 (0.015)
27	3	0.550 (0.008)	0.231 (0.018)	0.58 (0.06)	4297 (3691)	1.96 (1.00)	5989 (1663)	0.154 (0.029)
28	15	0.484 (0.033)	0.178 (0.022)	0.62 (0.11)	5072 (1875)	1.99 (1.04)	7247 (5225)	0.139 (0.034)
29	15	0.469 (0.063)	0.160 (0.019)	0.64 (0.08)	4816 (1397)	1.56 (0.59)	5496 (2462)	0.126 (0.031)
32	15	0.351 (0.106)	0.111 (0.064)	0.31 (0.11)	3207 (2085)	3.06 (1.79)	7928 (4561)	0.092 (0.032)

*Values are reported as Mean (SD)*.

**Figure 3:**
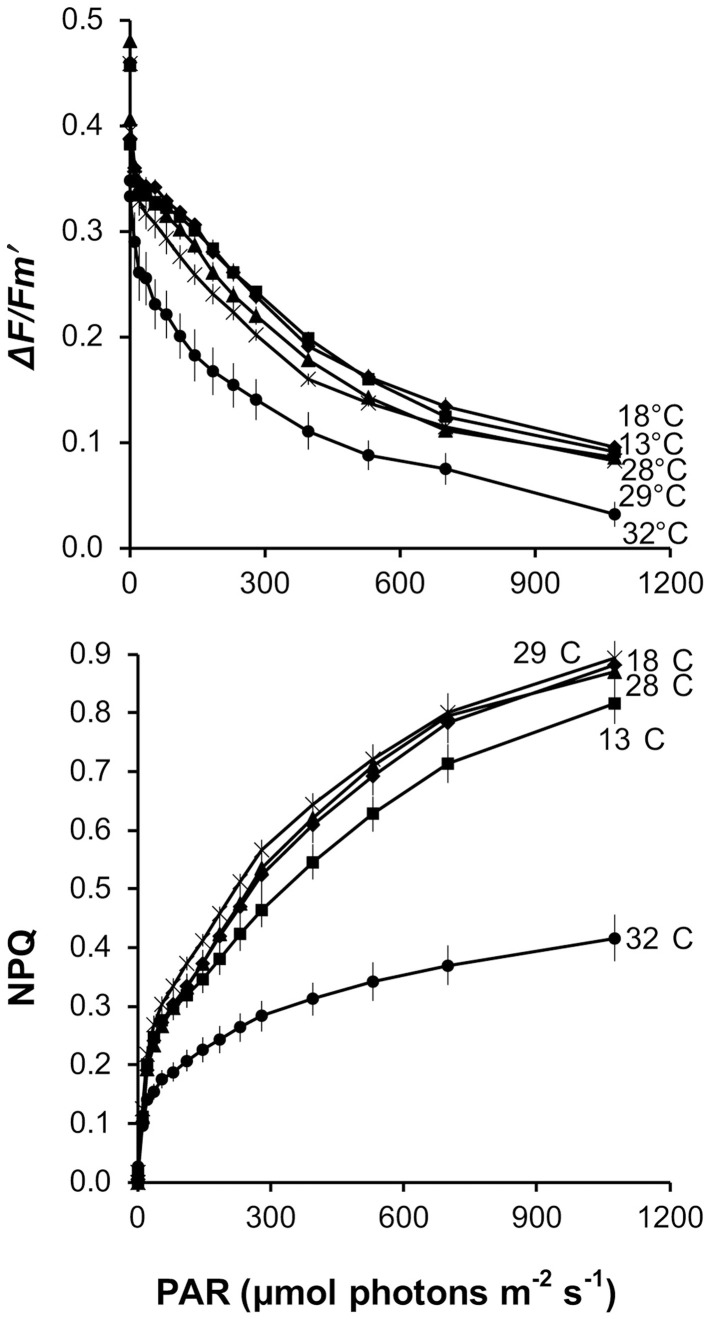
*****Balanophyllia europaea*****. Rapid light curves indicating Δ*F*/$F_{m}\prime$_(396)_ and non-photochemical quenching (NPQ) for each irradiance step at the five temperature treatments (13°C, squares; 18°C, diamonds; 28°C, triangles; 29°C, asterisks; 32°C, circles). Error bars are standard errors.

RLCs were obtained for all samples. Mean Δ*F*/$F_{m}\prime$ and NPQ for each temperature treatment and irradiance step are indicated in Figure 3. The highest Δ*F*/$F_{m}\prime$ was obtained at 18°C. It slightly decreased from 18 to 13°C, and decreased again from 13 to 28–29°C. Δ*F*/$F_{m}\prime$ at 32°C was notably lower than at all other temperature treatments (Figure 3). NPQ was maximum at 29°C. It slightly decreased at 18 and 28°C and decreased again at 13°C. At 32°C, NPQ was notably lower than at all other temperature treatments (Figure 3).

**Figure 4:**
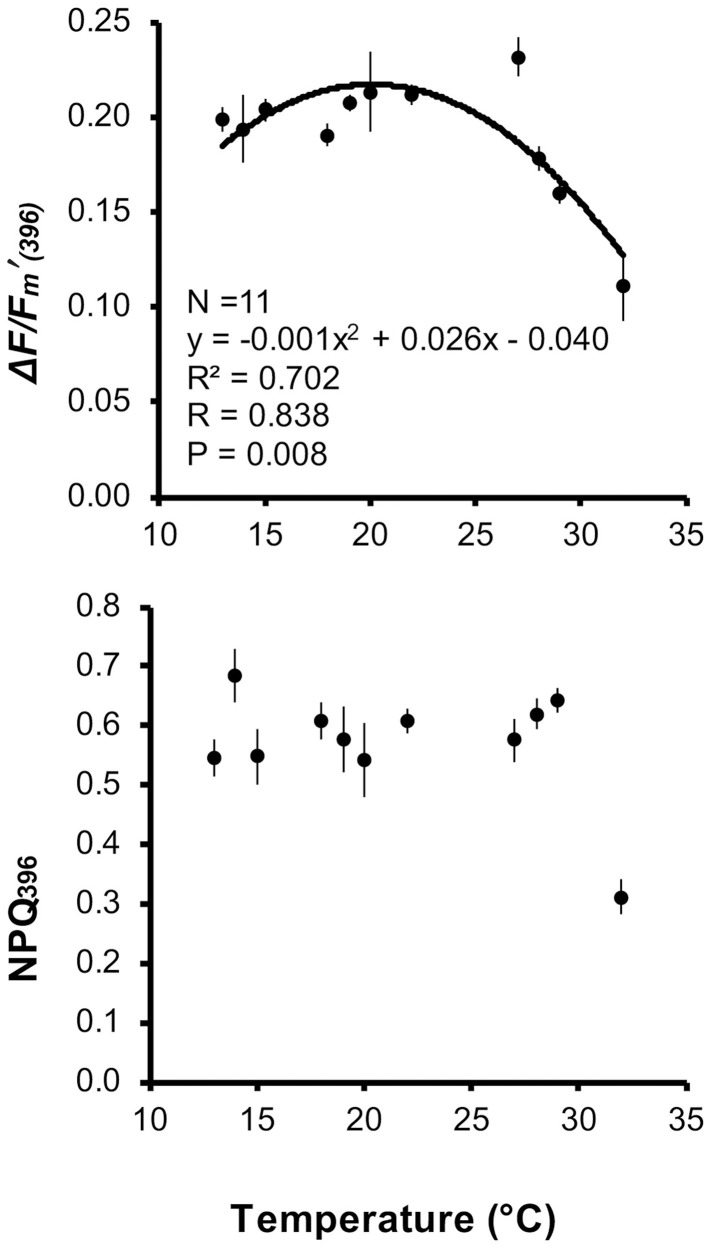
*****Balanophyllia europaea*****. Regression and correlation analysis between effective quantum yield at 396 PAR [Δ*F*/$F_{m}\prime$_(396)_], non-photochemical quenching at 396 PAR (NPQ_396_), and temperature using a quadratic function model. Error bars are standard errors. N number of temperature treatments.

Δ*F*/$F_{m}\prime$_(396)_ and NPQ_396_ were significantly different among temperature treatments (Kruskal-Wallis test/ANOVA, *P* < 0.001; Table 2). When fitted with a quadratic function, Δ*F*/$F_{m}\prime$_(396)_ was correlated with temperature (*P* < 0.001), whose variation explained 70.2% of Δ*F*/$F_{m}\prime$_(396)_ variance. According to the quadratic function, maximum Δ*F*/$F_{m}\prime$_(396)_ occurred at 20.0°C and while Δ*F*/$F_{m}\prime$_(396)_ decreased by 17.5% from 20.0 to 13°C, it decreased by 30.0% from 20.0 to 32°C (Figure 4). NPQ_396_ was not correlated with temperature (Figure 4). However, NPQ_396_ at 32°C was about 50% less than in the other treatments (Table 2).

**Figure 5:**
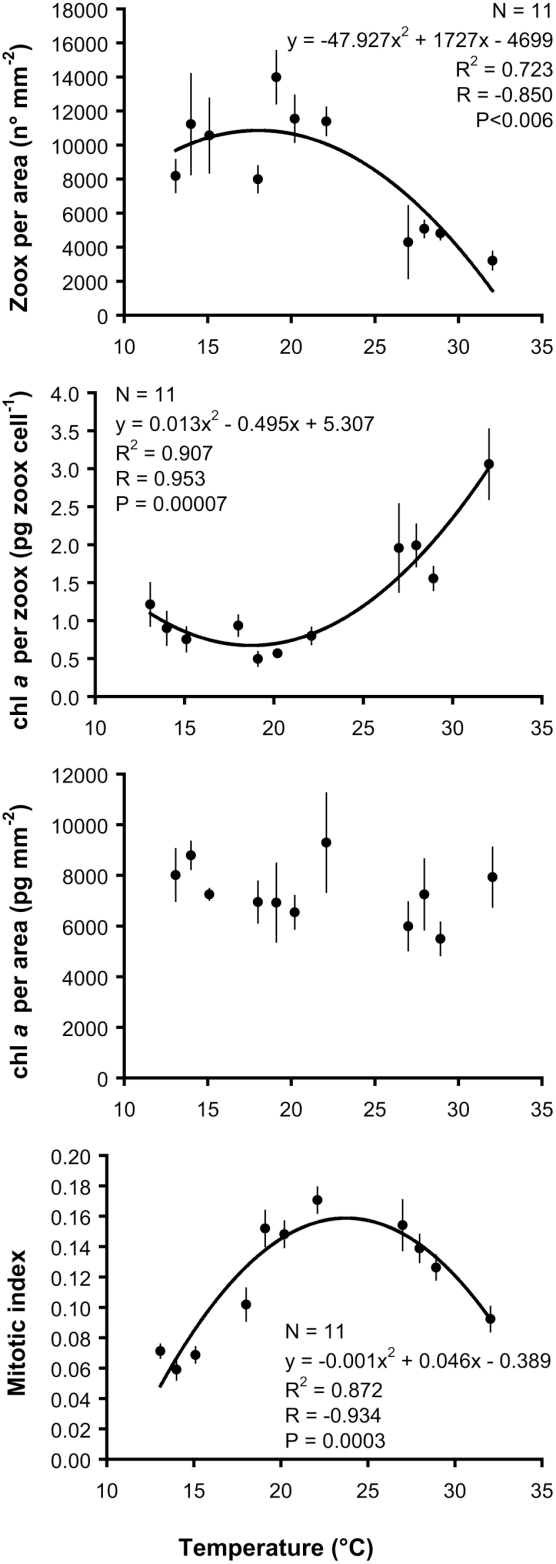
*****Balanophyllia europaea*****. Regression and correlation analysis between zooxanthellae number per area, chl *a* per zooxanthellae cell, chl *a* per area, mitotic index, and temperature using a quadratic function model. Error bars are standard errors. N number of temperature treatments.

Mean number of zooxanthellae per area, chl *a* content per zooxanthellae cell, chl *a* content per area, and mitotic index for each temperature treatment are indicated in Table 2. Mean zooxanthellae number per area and mitotic index were significantly different among temperature treatments (ANOVA, *P* < 0.001). While mean chl *a* content per zooxanthellae cell was significantly different among temperature treatments (Kruskal–Wallis test, *P* < 0.01), mean chl *a* content per area was homogeneous (Kruskal–Wallis test, *P* > 0.05). When fitted with a quadratic function, the number of zooxanthellae per area, the chl *a* content per zooxanthellae cell, and the mitotic index were significantly correlated with temperature, whose variation explained 72.3–90.7% of their variance (Figure 5).

## Discussion

Mean analysis day was homogeneous among temperature treatments, indicating that even if the corals from different treatments were analyzed in different days along 1 month, this did not bias the analysis since the samples from the five tanks were analyzed in homogeneous periods.

The indicators of photosynthetic performance analyzed consistently showed that the symbiotic system *B. europaea* reached its maximum photosynthetic efficiency of PSII within the temperature range 20.0–21.6°C (*F*_*v*_/*F*_*m*_ values suggest max efficiency at 21.6°C; Δ*F*/$F_{m}\prime$_(396)_ at 20.0°C). This was confirmed by the RLCs performed at the five temperature treatments, with the highest Δ*F*/$F_{m}\prime$_(396)_ at 18°C. Current seawater temperature at 6 m at Calafuria spans from 12 to 13°C in winter to peaks of 28°C in summer time, resulting in a mean SST of about 18°C (Goffredo et al., 2007, 2008, 2009). It then seems that the photosynthetic apparatus of *B. europaea* is adapted in performing better at temperatures slightly higher than the annual SST mean. This would imply that during winter and summer the coral experiences a considerable reduction of the opportunity for storing energetic resources coming from symbiotic algae via the photosynthesis. This energetic limitation is expected to be stronger during summer, since both for the *F*_*v*_/*F*_*m*_ and Δ*F*/$F_{m}\prime$_(396)_, the parameter values above the optimal temperature drop down to lower levels than those reached below the optimal temperature. Moreover, this effect is likely to be exacerbated in the field. In fact, during the present experiment, corals were regularly fed and never suffered from starvation. Instead, in the Mediterranean Sea, nutrients and zooplankton are typically lower during summer, due to thermal stratification leading to severe oligotrophy and plankton reduction, than in winter times when mixing and nutrient redistribution occur (Coma et al., 2000, 2009; Coma and Ribes, 2003). Low nutrients and zooplankton availability cause stress and starvation in the Mediterranean zooxanthellate scleractinian *Cladocora caespitosa* (Peirano et al., 2005) and summer dormancy in the metabolism of several benthic suspension feeders (Coma et al., 2000; Coma and Ribes, 2003). This consideration is in complete agreement with the timing of the annual reproductive cycle of *B. europaea* (Goffredo et al., 2002) with gonadal development taking place when zooplankton is more abundant (January-February). Furthermore, translocated photosynthates coming from the zooxanthellae have been termed as “junk food” suitable only for respiration, not for cell growth (Falkowski et al., 1984). In addition, in the tropical zooxanthellate corals *Stylophora pistillata* and *Galaxea fascicularis*, calcification and photosynthesis are lower in starved than in fed corals (Houlbrèque et al., 2004; Borell and Bischof, 2008; Borell et al., 2008). During winter, the decrease of energetic resources of *B. europaea* coming from photosynthesis may be counterbalanced by energy coming from heterotrophic feeding. In contrast, during summer, the depression of metabolic photosynthates, coupled with the deficiency of heterotrophic nourishment, is likely to cause a serious energetic deficit to *B. europaea* corals. This would explain the negative effects on several biological parameters (reduced skeletal density, population abundance, population structure stability, percentage of young individuals, calcification rate, and increased skeletal porosity) of the species in populations characterized by higher mean annual SST (Goffredo et al., 2007, 2008, 2009; Caroselli et al., 2011). However, it has to be noted that the use of PAM-fluorometry has several limitations (Enriquez and Borowitzka, 2010), such as the assessment of the physiological performance only of the algal partner of the coral-zooxanthellae symbiosis. The host could have several ways to interact with the response of the symbiont, through fluorescent pigments and mycosporine-like aminoacids for managing high light levels, increased heat shock protein expression to mitigate a cellular stress response, and elevated production of antioxidants (Baird et al., 2009). The response of the holobiont to high temperatures could also result from the stress directly impacting host cells (Paxton et al., 2013) or from the influence of skeletal architechture on light harvesting (Enriquez et al., 2005). Moreover, a thorough analysis of the adverse effect of elevated temperature on coral physiology also requires attention to the effect of temperature on respiration of both symbiotic partners, the net photosynthesis production, and photosynthesis:respiration ratio. Thus, further studies would be necessary to better characterize in detail the response of the *B. europaea* symbiotic system to high temperature, where the physiological responses of both partners of the symbiosys are analyzed.

Dissipation of light through non-photochemical pathways is an effective photoprotective mechanism in corals (e.g., Hoegh-Guldberg and Jones, 1999; Gorbunov et al., 2001), and other photosynthetic organisms under supra-optimal irradiance for which energetic costs of photoinhibition are apparent (e.g., higher plants and phytoplankton; Pahl-Wostl, 1992; Werner et al., 2001). Through NPQ, the excess absorbed light energy can be dissipated over both short (seconds to minutes) and long (tens of minutes to hours) time periods (Ruban and Horton, 1995). NPQ_396_ values were quite constant up to 29°C, suggesting that the *B. europaea* symbiotic system is quite well-adapted to the maximum light regime in the field even at temperatures 1°C higher than currently experienced. However, NPQ_396_ dropped abruptly by about 50% at 32°C, suggesting that at this temperature, a partial reduction of, and/or damage to, primary PSII acceptors will likely take place. The capability of high temperatures to make the coral–algal complex more susceptible to the damaging effect of solar radiation is well-documented (Brown, 1997; Hoegh-Guldberg, 1999; Fitt et al., 2001; Bhagooli and Hidaka, 2004). Capacity for photoacclimation and tolerance to high irradiance stress has been linked to the genetic type of *Symbiodinium*, both in culture and within multiple coral hosts (Robison and Warner, 2006; Warner et al., 2006). While the observed response of NPQ to temperature confirms the threat posed by the projected seawater temperature increase for the current century (Solomon et al., 2007) on the survival of *B. europaea* (Goffredo et al., 2007, 2008, 2009; Caroselli et al., 2011), it does not explain the decline in photosynthetic efficiency at temperatures higher than 20–21°C and lower than 32°C. If the decline in photosynthetic efficiency above 20–21°C was due to photoinhibition caused by high irradiance and temperature, one would expect a consequent increase of NPQ, which was not the case. The reduction of photosynthetic efficiency could thus be due to a detrimental effect of high temperature alone on the symbionts, host, or both, but further experiments are necessary to clarify the actual metabolic processes involved. The decrease in photosynthesis rates under elevated temperatures is most likely to be exacerbated by the steep increase in both host and symbiont respiration rates, which were not measured here. However, there is ample documentation of the steep dependence of plant, algal and coral respiration on temperature, both in adult colonies (e.g., Jokiel and Coles, 1990) and in planulae (Edmunds et al., 2011).

The number of zooxanthellae per area was maximum around 18°C, decreased from 18 to 13°C and strongly decreased from 18 to 32°C (Figure 5). At the same time, chlorophyll concentration per zooxanthellae cell displayed an opposite trend, resulting in homogeneous chlorophyll content per area among temperature treatments (Figure 5). This response has been put in relation with space limitation and self-shading of zooxanthellae cells (Hoegh-Guldberg and Smith, 1989; Fitt et al., 1993; Trench, 1993; Jones, 1997; Jones and Yellowlees, 1997; Stambler and Dubinsky, 2005; Hoogenboom et al., 2006) and with nutrient limitation (Muscatine et al., 1989; Houlbrèque et al., 2004). While nutrient limitation could be excluded, since corals were regularly fed (however, see Muscatine et al., 1989), at low zooxanthellae density, self-shading would be prevented, and an increase of chlorophyll content per zooxanthellae cell could be advantageous for recolonization of host tissue by zooxanthellae cells after the temperature stress. The observed pattern is different with what reported for the Mediterranean *C. caespitosa* exposed to short-term high temperatures, showing a decrease in chlorophyll per zooxanthellae cell and no decrease in zooxanthellae density (Rodolfo-Metalpa et al., 2006). The low mitotic index observed at high temperatures may indicate the inability of stressed zooxanthellae to recolonize host tissue even at low zooxanthellae density (Jones and Yellowlees, 1997). This could be determined by host factors regulating the dynamics of the zooxanthellae population in the host (Baghdasarian and Muscatine, 2000; Stambler, 2011). In fact, some coral species preferentially expel dividing zooxanthellae cells when the host cannot tolerate an increase of its symbionts population (Baghdasarian and Muscatine, 2000). Since at high temperatures and light (as in summer) the algal cells are likely to increase the levels of reactive oxygen species that would be detrimental for the host (Lesser, 2006), the coral may opt to expel most of its symbionts, especially the dividing cells (Suharsono and Brown, 1992; McCloskey et al., 1996; Baghdasarian and Muscatine, 2000). This would explain the observed reduction of mitotic index at high temperatures, but further studies are needed to clarify this aspect.

In conclusion, the optimal temperature for photosynthesis of the symbiotic system of *B. europaea* is slightly higher (20.0–21.6°C) than the annual mean the corals experienced in the field (18°C). At temperatures >21.6°C, all parameters analyzed showed a reduction of photosynthetic efficiency, up to the loss of zooxanthellae cells and consequent bleaching. Even if this study has the limitation of focusing only on the algal partner, it strengthens the hypothesis that the negative effects of high temperatures on this species reported in literature (Goffredo et al., 2007, 2008, 2009; Caroselli et al., 2011) are caused by a reduction of photosynthetic performance and consequent decline of the energetic resources available for the coral. The temperature driven decrease in photosynthesis, coupled with the possible limitation in nutrient and plankton availability in summer (Coma et al., 2000, 2009; Coma and Ribes, 2003) combine to a dire forecast for the future of *B. europaea*. The results obtained here on *B. europaea* suggest that increasing research efforts on the poorly studied temperate corals will be important to investigate their potentially negative responses to the predicted rate of increasing seawater temperature.

## Author contributions

GF, SG, ZD, and OL conceived and designed the experiment. EC performed the experiments. All authors wrote the manuscript and participated to the scientific discussion.

### Conflict of interest statement

The authors declare that the research was conducted in the absence of any commercial or financial relationships that could be construed as a potential conflict of interest.
